# The Optimization of the Luminescence Intensity Ratio in the YVO_4_:Er^3+^,Nd^3+^ Phosphors within the First and Second Biological Windows

**DOI:** 10.1021/acsomega.5c10803

**Published:** 2026-01-21

**Authors:** Francisca J. R. Tavares, André S. Laia, Matheus S. C. de Oliveira, Ariosvaldo J. S. Silva, Nilson S. Ferreira, José J. Rodrigues, Márcio A. R. C. Alencar, Marcos V. dos S. Rezende

**Affiliations:** 1 Functional Nanomaterials Group (GNF), Department of Physics, 74391 Federal University of Sergipe, São Cristóvão, SE 49100-000, Brazil; 2 Department of Physics, Federal University of Sergipe (UFS), São Cristóvão, SE 49100-000, Brazil; 3 Corrosion and Nanotechnology Laboratory (LCNT), Federal University of Sergipe (UFS), São Cristóvão, SE 49100-000, Brazil; 4 Research Group in Physics and Physics Education (GPFEF), Department of Natural Sciences, State University of Pará, Marabá, PA 68502-100, Brazil; 5 Graduate Program in Physics, Institute of Physics, Federal University of Mato Grosso, Cuiabá, MT 78060-900, Brazil

## Abstract

The optimization of the host materials is one of the methods that could be used to improve the relative sensitivity of luminescent thermometers. Here, we show the host optimization emission of Er^3+^ and Nd^3+^ ions in the YVO_4_ phosphors produced by a modified sol–gel route using glucose as a polymerizing agent. X-ray diffraction (XRD) was realized to analyze the formation of the crystalline phase. We found that the emission ratio depends on the size of the YVO_4_ particle. Here, we show that YVO_4_:Er^3+^,Nd^3+^ phosphors exhibit particle size-dependent relative sensitivity values, varying from 1.32 ± 0.02 to 1.80 ± 0.02% K^–1^ at 293 K, and temperature uncertainties below 0.7 K within the physiological range 293–233 K, operating entirely in the first and second biological windows. These sensitivity and temperature uncertainty values were achieved by defining two thermometric parameters as the luminescence intensity ratios, exploiting a combination of emissions from both ion species.

## Graphical abstract

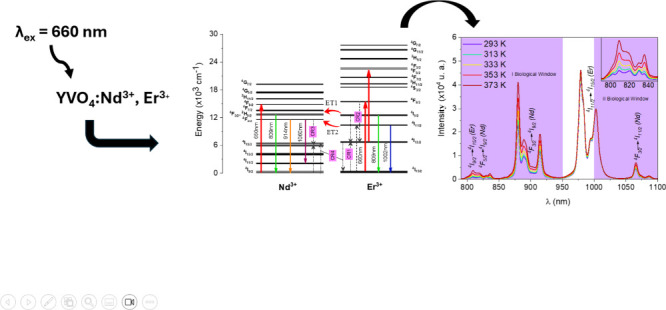

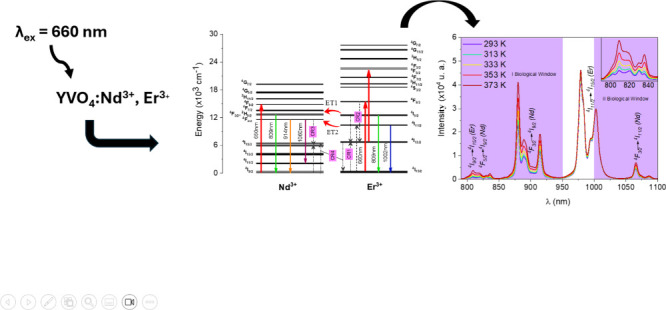

## 1 Introduction

The search for accurate and reliable optical temperature sensors has grown intensively in recent years.^1–8^ Due to their invulnerability to environmental conditions and strong electromagnetic fields, these sensors can be applied to very specific situations in which conventional techniques are not suitable. Moreover, optical temperature sensors can perform real-time measurements and employ nanometric probes, such as luminescent nanoparticles, which are necessary features for applications that require high spatial resolution and sensitivity.^5,6,8–10^

For instance, exploiting the temperature-dependent luminescence of nanophosphors, it has been possible to employ optical temperature sensors in a myriad of applications, monitoring temperature of electronic and photonic devices, and biological systems as well.^5,11^ For the latter, it has been demonstrated that luminescence nanothermometry is a very powerful ally to nanomedicine, playing a key role in the development of both diagnosis and therapeutical procedures.^4,5,7,11,12^

One of the main requirements for the development of luminescence temperature sensors aiming at *in vivo* applications is to make them as less invasive as possible. An efficient strategy used to achieve this purpose is the design of systems that allow remote temperature reading. For that, the excitation source and the emission wavelengths are properly chosen to lie within the electromagnetic spectrum regions called biological windows (BWs), in which light scattering due to the biological tissue is minimized. Three of those are in the visible and near-infrared regions: BW I, from 650 to 950 nm; BW II, from 1000 to 1350 nm; and BW III, from 1500 to 1850 nm.^13,14^ In addition, the luminescent probe should: exhibit efficient absorption and emission of light, presenting high brightness without the need of high excitation power, which prevents damage to biological tissues; exhibit long-term photostability; exhibit high thermal sensitivity; and present low toxicity in living organisms. Finally, combining all those features, the designed system must present fast temporal response and satisfactory temperature and spatial resolutions.^8,10,13,14^

Nevertheless, the development of luminescent temperature sensors with good accuracy, allowing noninvasive temperature measuring and mapping, is still a challenge. Phosphors based on different classes of inorganic, organic, and hybrid materials have been proposed in recent years with this propose, due to their luminescent properties and high-temperature sensitivity.^15^ Among them, it has been demonstrated that yttrium vanadate (YVO_4_) exhibits a huge potential as an optical temperature probe when doped with luminescent ions. It has a large crystal structure, with excellent thermal, optical, mechanical, and chemical properties. It is not easily photodegraded, and it has low toxicity.^8,9,16^ It has a relatively low phonon energy of 880 cm^–1^ and exhibits good upconversion efficiency when doped with rare earth ions.^5,17^ It was also reported that the particle size and morphology of YVO_4_ are influenced by the temperature calcination and synthesis methods.^18,19^ In the near-infrared region, YVO_4_ doped with Nd^3+^ ^5,20,21^ and codoped with Nd^3+^ and Er^3+^ particles^22^ have been investigated as temperature sensing probes aiming applications within the I and II biological windows and the influence of dopant concentration on the sensitivity was also evaluated.

Nevertheless, according to the probe luminescence features, different approaches can be used in the sensor development, exploiting different temperature-dependent mechanisms.^1^ For temperature sensor probes based on a solid-state transparent host doped with rare earth ions, one of the most used techniques for the analysis of optical temperature sensors is the luminescence intensity ratio (LIR). This methodology was originally proposed based on the relative change of the emission intensities associated with transitions from two thermally coupled levels. In this case, the energy gap between the emitter levels varies from 200 to 2000 cm^–1^ and they are in thermodynamic quasi-equilibrium.^3,4,16,17,23^ Alternatively, the same methodology can be applied to pairs of levels that are not coupled thermally (nonthermally coupled levels), whose population densities are correlated by temperature through nonradiative transitions.^3,4,16,17,23^ Moreover, the sensitivity of this technique is strongly correlated with the choice of the dopants and the structure and morphology of the nanometric host.^18,19^

Indeed, it was observed that the relative sensitivity decreases with the increase of the YVO_4_:Yb^3+^, Er^3+^ particle size when the LIR temperature sensing scheme is based on the thermal coupling between the ^4^S_3/2_ and ^2^H_11/2_ levels of Er^3+^.^18^ Surprisingly enough was the result reported for the influence of the particle size on the absolute sensitivities of Er^3+^/Yb^3+^ codoped Y_2_O_3_ microspheres exploiting thermally coupled and uncoupled levels.^24^ Exploiting the same thermally coupled pair of levels as in ref 18, the absolute sensitivity increased with the particle size, while the size influenced only slightly the relative sensitivity. On the other hand, the LIR scheme was used based on the luminescence from two nonthermally coupled levels of Er^3+^ (^2^F_9/2_ /^2^H_11/2_); both sensitivities decreased with the increase of the particle size. The effect of the particle size on the thermometric performance of single doped Y_2_O_3_:Er^3+^ particles have also been investigated.^12^ Using the same thermally coupled LIR scheme as in refs 18,24 , the observed sensitivity behavior with the particle size was the opposite, while, exploiting the emission from two nonthermally coupled levels (^4^S_3/2_ and ^4^F_9/2_), the relative sensitivity was higher for smaller nanoparticles. The influence of the calcination temperature on the relative sensitivity was also investigated using Y_2_O_3_:Er^3+^ ^25^ exploiting LIR based on emissions in the near-infrared region from nonthermally coupled levels (^4^I_11/2_, ^4^F_9/2_, ^4^I_9/2_, and ^4^S_3/2_). For all investigated pairs of levels, the sensitivity behaviors are influenced by the temperature calcination but in different ways. A similar investigation was conducted exploiting Y_2_O_3_ particles doped with Nd^3+^.^26^ In this case, single band emission schemes were exploited. It was observed that the micrometric particles presented a better performance than the nanometric ones. The effect of the nanoparticle size on the temperature sensing and optical heating performance in Ho^3+^/Tm^3+^-codoped KLu­(WO_4_)_2_ nanoparticles^27^ was also reported. The author observed that particles with smaller sizes generated heat more efficiently, but their capacity to sense temperature was inferior in comparison to that of the agglomerated nanocrystals. These examples highlight the richness of schemes that can be exploited for temperature sensing and the need to evaluate the influence of the particle size for each proposed sensing scheme aiming its optimization.

In this work, we investigated the performance of YVO_4_:1%Er^3+^,1%Nd^3+^ nanocrystals as temperature probes operating within BW I and BW II, prepared by the modified sol–gel synthesis route, varying the calcination temperature. The morphology and crystalline structure of the produced particles were analyzed using transmission electron microscopy (TEM) and X-ray diffraction (XRD). The samples were characterized as temperature sensors using a laser emitting at 660 nm as the excitation source and exploiting LIRs within BW I and BW II. The thermometric performances of all samples were evaluated in terms of relative sensitivity, reproducibility, repeatability, and temperature uncertainty.

## 2 Methodology

The quantities of the initial salts were varied following the formula YVO_4_:1% Er^3+^,1% Nd^3+^; they were synthesized via a modified sol–gel route using glucose as a chelating agent. Precursor solutions with the following reagents were used: Y­(NO_3_)_3_·6H_2_O (Sigma-Aldrich, 99.8%), NH_4_VO_3_ (G-cec, 99%), Er­(NO_3_)_3_·5H_2_O (Sigma-Aldrich, 99.9%), Nd­(NO_3_)_3_·6H_2_O (Sigma-Aldrich, 99.9%), and anhydrous glucose. The stoichiometric balance of the reagents was performed, and then they were weighed on a precision balance. The initial reagents were added to a solution composed of 20 mL of distilled water and a glucose molar ratio equal to 1:2 (YVO_4_/glucose). The solution was then stirred and heated to 150 °C for 2 h on a magnetic heating plate to eliminate water until a gelatinous solution was formed. Finally, the xerogels were calcined at different temperatures (900, 1000, and 1100 °C for 2 h) under air to form a powder, which was then kneaded and homogenized to obtain the final product. X-ray diffraction (XRD) techniques were used to analyze the formation of the crystalline structure and its structural properties. XRD was obtained in a 2θ range of 15 to 80°, using the Panalytical Empyrean Series 2 powder diffractometer, which uses a Cu tube with Cu Kα = 1.5419 Å radiation. Scanning electron microscopy (SEM) images were acquired using a Helios 5 PFIB CXe DualBeam microscope, using a voltage of 2 kV, at the Microscopic Samples Laboratory (LAM) of the National Synchrotron Light Laboratory (Sirius-LNLS), Campinas-SP, Brazil.

The samples studied were pressed into an aluminum sample holder, occupying a circular surface 4 mm in diameter. This sample holder was placed on a heating plate with controlled temperature (between 293 and 373 K), and the temperature was monitored by a thermocouple, with a resolution of 1 °C, positioned about 2 mm from the sample. The samples were excited by a CW laser diode emitting at 660 nm, with a power of 10 mW. A bandpass filter (FB660 ± 10—Thorlabs) was used to ensure monochromatic laser emission. The sample emission was collected by a lens array and guided through an optical fiber to a compact CCD spectrometer (Maya2000-Pro—Ocean Optics). Two bandpass filters (FGL710—Thorlabs) were used at the fiber input to reject the excitation light. Power sweep tests were performed by controlling the laser power by using neutral filters.

## 3 Results and Discussion

Figure 1 shows the X-ray diffraction patterns of the prepared YVO_4_:1%Er^3+^,1%Nd^3+^ samples calcined at 900, 100, and 1100 °C. All peaks of all samples match with the standard card of tetragonal YVO_4_ (space group *I*4_1_/*amd*, ICSD No. 02504). A slight uniform left shift relative to ICSD is due to a small instrumental zero shift or sample displacement and is fully corrected in the Rietveld fits, shown in Figure 2. No changes in the positions of the diffraction peaks are observed due to the Er and Nd dopant ions. This indicates that these ions were effectively incorporated into the YVO_4_ lattice. The incorporation of Er and Nd did not affect the diffraction line positions because of the low dopant concentrations used and the similar ionic radii of the dopants and the Y sites in the host lattice. The peak positions also remain unchanged with variations in the calcination temperature. A slight decrease in the full width at half-maximum (fwhm) is observed as the calcination temperature increases. Figure 2 shows the fwhm of the six strongest reflections (2θ ≈ 25.0, 33.6, 35.7, 49.9, 62.8, and 75.7°). The 2θ values correspond to the centroid of each peak. The fwhm decreases slightly from 900 to 1100 °C. These results indicate that increasing the calcination temperature improves the sample’s crystallinity and increases the average crystallite size of YVO_4_.

**Figure 1:**
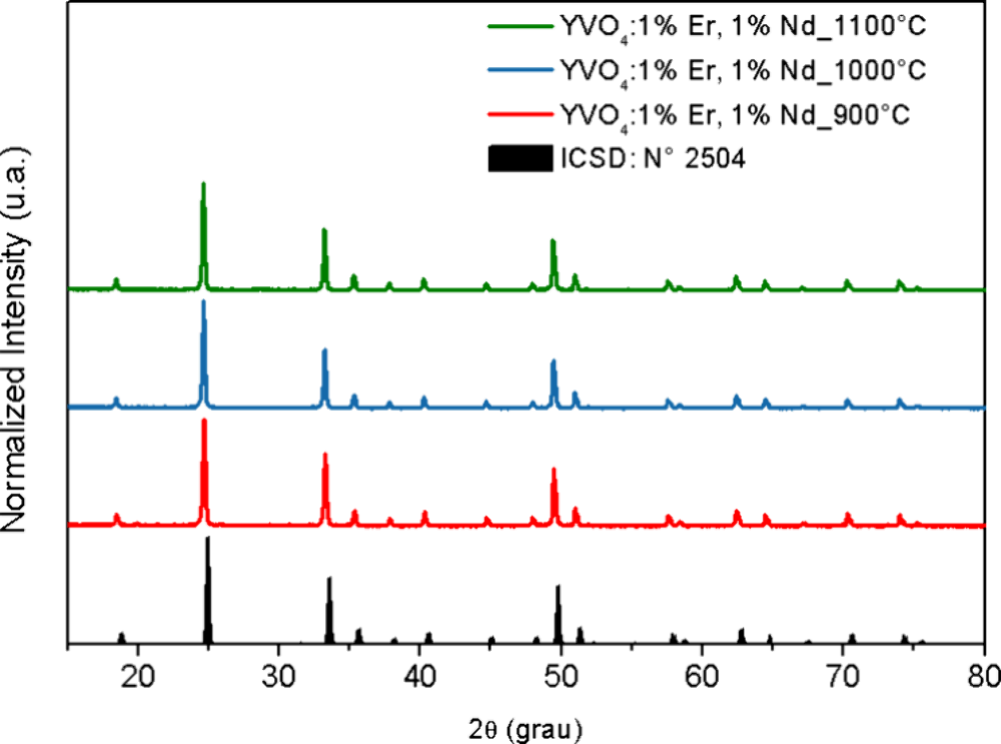
XRD spectra of samples of pure YVO_4_ and YVO_4_:1%Er,1%Nd calcined at 900, 1000, and 1100 °C.

**Figure 2:**
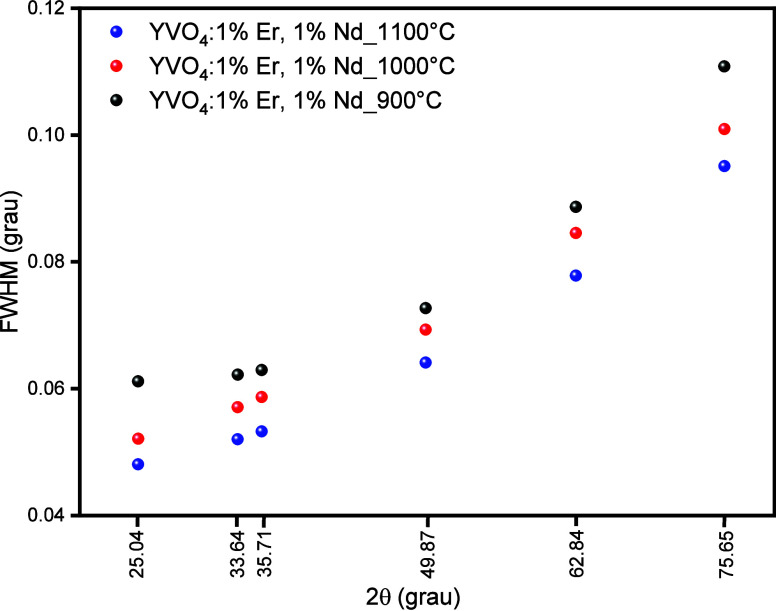
fwhm as a function of 2 for the six main XRD reflections of YVO_4_:1%Er,1%Nd calcined at 900, 1000, and 1100 °C. The 2θ values correspond to peak centroids determined from Figure 1.

Figure 3 shows structural refinement of the produced materials. This was performed adopting the atoms’ initial positions from those reported in the ICSD No. 02504 pattern. For all samples, the calculated XRD patterns matched well with their experimental counterparts. The obtained fit parameters are shown in Table 1. It is possible to note that the lattice parameters decreased slightly when the calcination temperature was increased from 900 to 1000 °C (Table 1).

**Figure 3:**
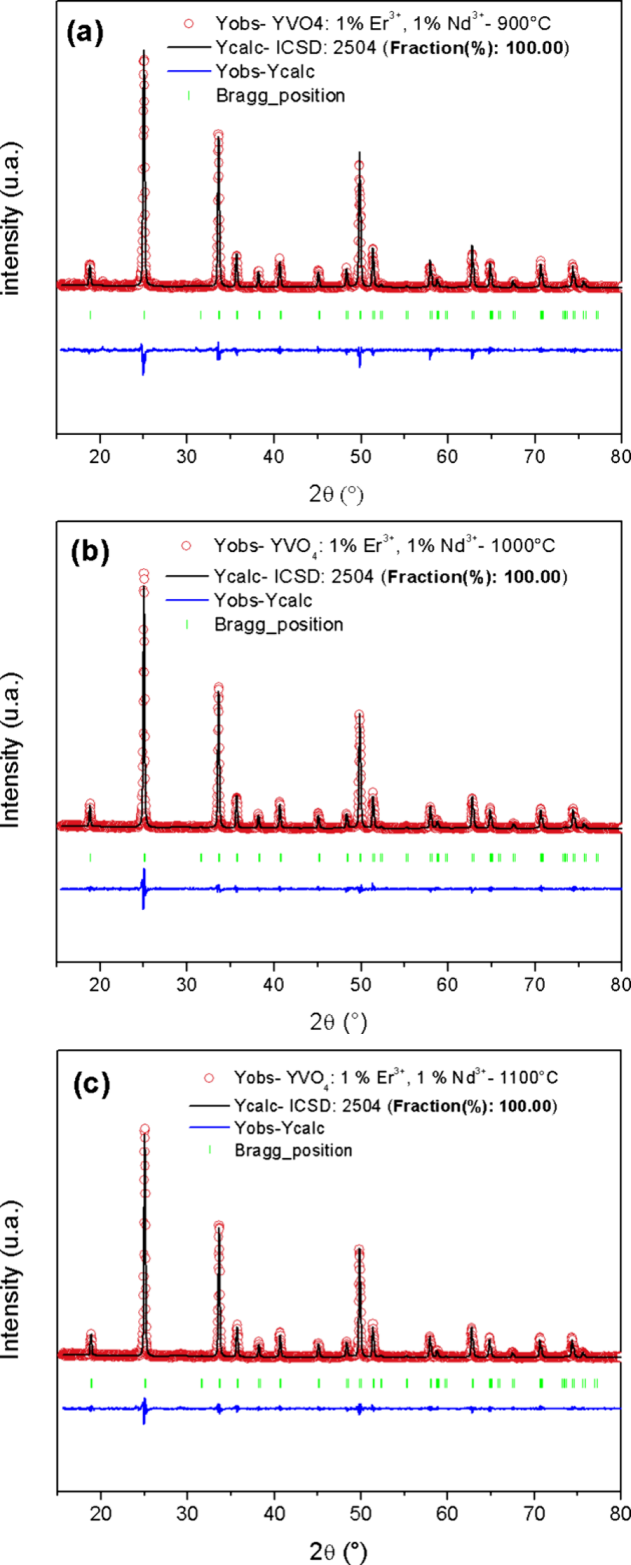
Structural refinement of YVO_4_:1% Er,1% Nd calcined at (a) 900, (b) 1000, and (c) 1100 °C.

**Table 1:** Calculated Lattice Parameters and Data Fit Parameters of the Prepared YVO_4_:1%Er^3+^,1%Nd^3+^ samples calcined at 900, 1000, and 1100 °C

samples	900 °C	1000 °C	1100 °C
*a* = *b* (Å)	7.11112 ± 0.00010	7.11110 ± 0.00010	7.10969 ± 0.00010
*c* (Å)	6.28271 ± 0.00010	6.28270 ± 0.00009	6.28323 ± 0.00009
*V* (Å^3^)	317.705 ± 0.008	317.527 ± 0.008	317.603 ± 0.008
χ^2^	1.55	1.24	1.39
*R*_F_	6.28%	3.34%	4.35%
*R*_B_	3.91%	3.67%	5.06%

Figure 4 shows the SEM images and particle size distribution of the YVO_4_:1%Er,1%Nd samples. The particle sizes were calculated by using a log-normal distribution function. It can be observed that the sample consists of agglomerated particles and that the particle sizes for YVO_4_:1%Er^3+^,1%Nd^3+^ samples under the calcination temperatures of 900, 1000, and 1100 °C were about 171 ± 3, 262 ± 11, and 309 ± 14 nm, respectively. The size of the particles tends to increase with increasing temperature due to diffusion in the solid state between the NPs in contact in the particle cluster, providing the connection of these NPs to form larger particles. This increase in particle size identified in the SEM images corroborates the increase in crystallinity identified from the increase in the intensity of the diffraction peaks presented in Figure 1, as the calcination temperature increases.^28^

**Figure 4:**
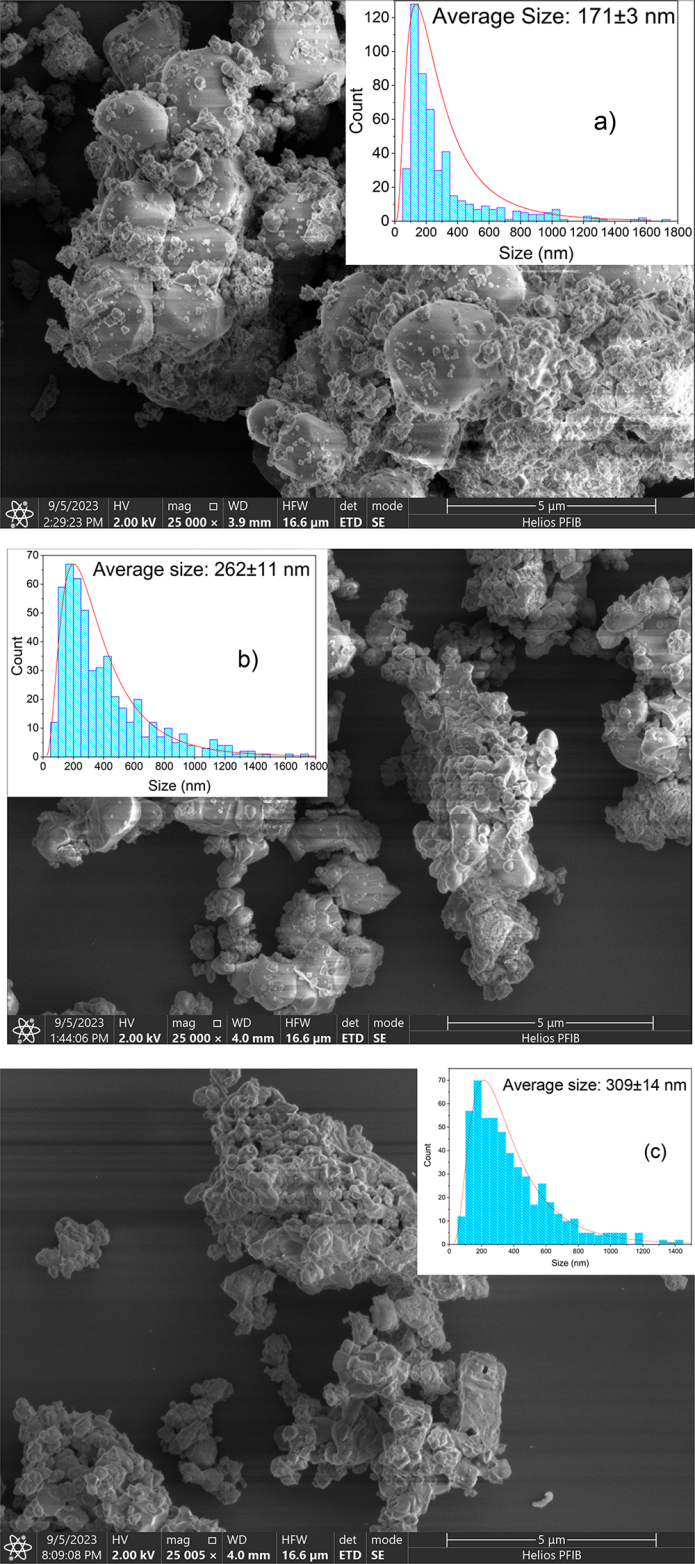
SEM images of YVO_4_:1%Er,1%Nd calcined at (a) 900 °C, (b) 1000 °C, and (c) 1100 °C.

It is known that luminescent properties vary with the size of the nanoparticles.^28^ To understand the influence of the grain size of the YVO_4_:1%Er,1%Nd on their luminescent properties, in Figure 5, the emission spectra of the samples calcined at 900, 1000, and 1100 °C are presented, measured at room temperature, and excited at λ_ex_ = 660 nm. It can be observed that as the calcination temperature increases, there is an increase in the overall luminescent intensity. This behavior can be associated with the increase of the particle size with the calcination temperature. Indeed, the smaller the particles, the more important the contribution of rare earth ions on the particle surface to the overall luminescence. As the ions located closer to the surface are more susceptible to the influence of external nonradiative decay paths, the luminescence of smaller particles is usually less intense than the light emission from larger particles.^25,29^ Indeed, the sample calcined at 900 °C presents the smallest average particle size and a larger number of particles smaller than 200 nm in comparison with the other two samples. Owing to that, the surface quenching effect is stronger, and the emission intensity is significantly smaller for the sample calcined at 900 °C. On the other hand, for the samples calcined at 1000 and 1100 °C, most particles are larger than 200 nm. For those, the influence of the surface effect on the luminescence is negligible, and despite the difference on the particle average size, their luminescence intensities are similar. Variations with calcination temperature lead to larger particles and to small changes in a, c, and c/a (Table 1). These changes imply a slight modulation of the crystal field at the Nd^3^^+^ site through minor adjustments of the Nd–O distances and local symmetry. No resolvable Stark shifts were observed.^30,31^ The increase in emission with temperature is mainly due to particle growth and to the reduced surface-to-volume ratio, which mitigates nonradiative surface quenching.

**Figure 5:**
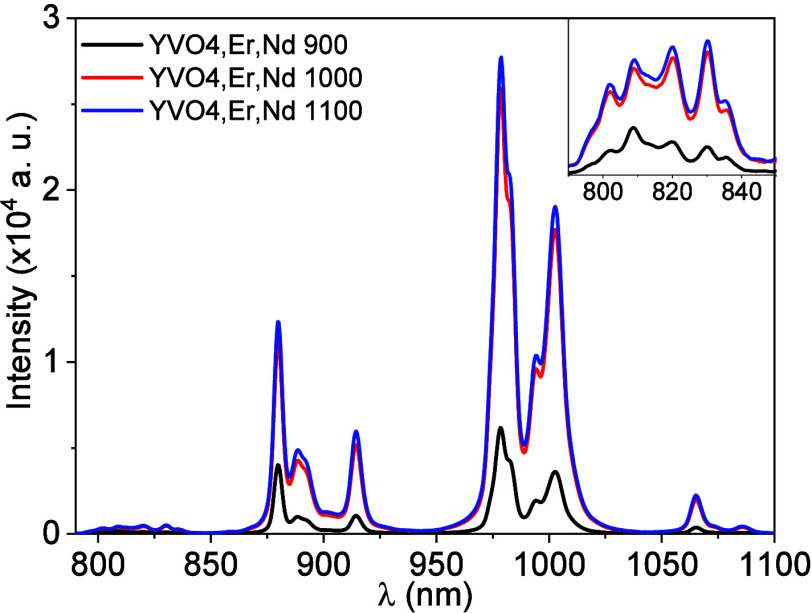
Emission spectra for the YVO_4_:1%Er, 1%Nd sample annealed at different temperatures (λ_ex_ = 660 nm).

Figure 6 shows the temperature effect on the emission spectra of the YVO_4_:Er^3+^,Nd^3+^ (1 mol %, 1 mol %) sample calcined at 1100 °C when excited at 660 nm. The temperature varied within the range of 293–373 K. The emission spectrum in the infrared region (from 780 to 1100 nm) depends on the temperature, and the emission intensities change significantly in the 293–373 K range. This behavior indicates that this system can be used to measure the temperature in I-BW and II-BW.

**Figure 6:**
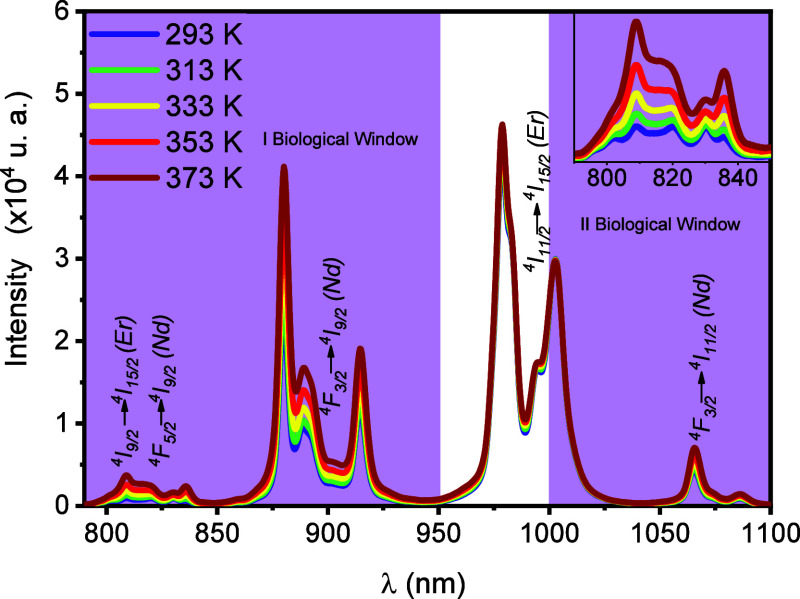
Emission spectra of YVO_4_:1%Er,1%Nd obtained at different temperatures (λ_ex_ = 660 nm).

The emission spectra consist of four emission bands localized at around 820 nm (Er^3+^:^4^I_9/2_-^4^I_15/2_, Nd^3+^:^4^F_5/2_-^4^I_9/2_), 880 nm (Nd^3+^:^4^F_3/2_-^4^I_9/2_), 975 nm (Er^3+^:^4^I_11/2_-^4^I_15/2_), and 1064 nm (Nd^3+^:^4^F_3/2_-^4^I_11/2_). As shown schematically in Figure 6, upon 660 nm irradiation, the electrons are optically excited from the ground state of Er^3+^ (^4^I_15/2_) and Nd^3+^ (^4^I_9/2_) ions up to its excited state (Er^3+^:^4^F_9/2_, Nd^3+^: ^4^F_9/2_), which is followed by the fast nonradiative decays to the ^4^I_9/2_, ^4^I_11/2_, and ^4^I_13/2_ of Er^3+^ and ^4^F_5/2_ and ^4^F_3/2_ of Nd^3+^ and subsequent radiative transitions to the ^4^I_15/2_ level of Er^3+^ and ^4^I_9/2_ and ^4^I_11/2_ levels of ions (Figure 6). Due to the host crystalline field, the ^4^F_3/2_ level of Nd^3+^ ions can be split into two Stark’s sublevels, while the lower energetic level, ^4^I_9/2_, is divided into five sublevels.

One can see that a temperature increase led to the increase of the emission intensities associated with the ^4^I_9/2_–^4^I_15/2_ transitions of Er^3+^, as well as the ^4^F_5/2_–^4^I_9/2_ and ^4^F_3/2_–^4^I_9/2_ transitions of Nd^3+^ ions. Nevertheless, the temperature affects the emission intensity differently due to the ^4^I_11/2_–^4^I_15/2_ transition of Er^3+^ ions. Owing to these behaviors, we proposed utilizing two different LIRs to measure temperature using this material as a sensing probe: LIR1 = *I*_809_/*I*_914_ and LIR2 = *I*_809_/*I*_1002_.

As previously described, the emission at 809 nm is composed of a combination of light emitted from both erbium and neodymium ions due to the transitions Er^3+^:^4^I_9/2_–^4^I_15/2_ and Nd^3+^:^4^F_5/2_–^4^I_9/2_. The emission at 914 nm, on the other hand, is associated with the Nd^3+^:^4^F_3/2_–^4^I_9/2_. Nevertheless, in our previous paper,^22^ we demonstrated that the emission peak at 809 nm is dominated by Nd^3+^ ion emissions, which increases with temperature. Although the emission lines at 809 and 914 nm also originate from the same level of Nd^3+^, they are associated with different Stark sublevels of the emitter level (^4^F_3/2_ and ^4^F_3/2_) and therefore have a different temperature dependence.

Thus, the LIR1 data exhibit a behavior closer to that of a thermally coupled system (^4^F_5/2_–^4^F_3/2_) whose energy gap when inserted into YVO_4_ is approximately 1000 cm^–1^.^30^ Therefore, LIR1 is proportional to the population density ratio of the thermally coupled levels of the Nd^3+^ ions, ^4^F_5/2_ and ^4^F_3/2_, which follows the Boltzmann distribution:^32^

$$\mathrm{LIR}1\left(T\right)=\frac{I_{809}}{I_{914}}=\frac{A_{2}hv_{2}N_{2}}{A_{1}hv_{1}N_{1}}=\frac{A_{2}hv_{2}N_{2}g_{2}}{A_{1}hv_{1}N_{1}g_{1}}B\exp\left(-\frac{\Delta E_{21}}{kT}\right)$$ 1

In this equation, *i* = 1 (2) corresponds to the ^4^F_3/2_ (^4^F_5/2_) level of Nd^3+^; *N*_*i*_ is the population density of level *i*, *v*_*i*_ is the frequency associated with the emission from level *i*, *A*_*i*_ is the total spontaneous emission from level *i*, *g*_*i*_ is the degenerescence of level *i*, Δ*E*_21_ is approximately the energy gap between the emitting levels, *k* is the Boltzmann constant, and *T* is the environment temperature.

This equation can also be written in terms of the ln­(LIR1) as

$$\ln\left[\mathrm{LIR}1(T)\right]=\beta -\frac{\alpha }{T}$$ 2

in which β = ln­(*B*).^32^

In the case of LIR2, while the emission at 809 nm is predominantly correlated with the luminescence of Nd^3+^ ions, *I*_1002_^Er^ is related to the ^4^I_11/2_–^4^I_15/2_ transition of Er^3+^ ions. As the bands corresponding to the intensities of 809 and 1002 nm originate from the different emitter levels, their dependences on temperature are essentially different and, therefore, both points were selected for temperature sensing analysis. Thus, the intensity ratio obtained is predominantly from the emissions of two distinct ionic species, which are not thermally coupled but are correlated by temperature. In this case, we observe that LIR2 exhibits an exponential behavior with temperature^22,31^

$$\mathrm{LIR}2\left(T\right)=C\exp(R_{0}T)$$ 3

in which *C* and *R*_0_ are fitting parameters.

Figure 7 presents the LIR1 and LIR2 results for the YVO_4_:1%Er,1%Nd samples calcined at 900, 1000, and 1100 °C. The luminescence experiments were carried out for the environment temperature varying between 293 and 373 K. For LIR1, the data are presented using a monologarithm scaled plot. It can be observed that LIR1 displays a linear decrease with the inverse of the environment temperature. Using eq 3, the thermometric parameters of this temperature sensor were obtained from the fitting parameters. For LIR2, the data are plotted by using a linear scale and exhibit an exponential increase with temperature. Using eq 4, the thermometric parameters of this temperature sensor were obtained from the fitting parameters (Figure 8).

**Figure 7:**
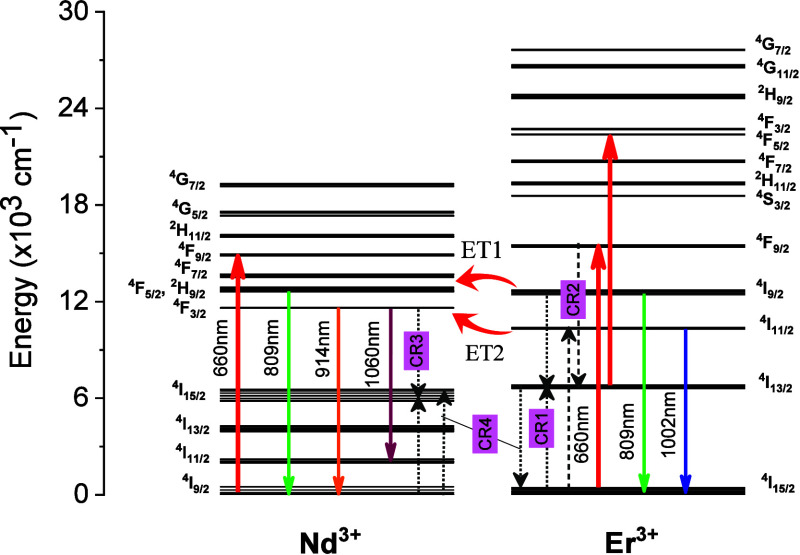
Thermal sensing scheme based on emission spectra of the Er^3+^ and Nd^3+^ ions.

**Figure 8:**
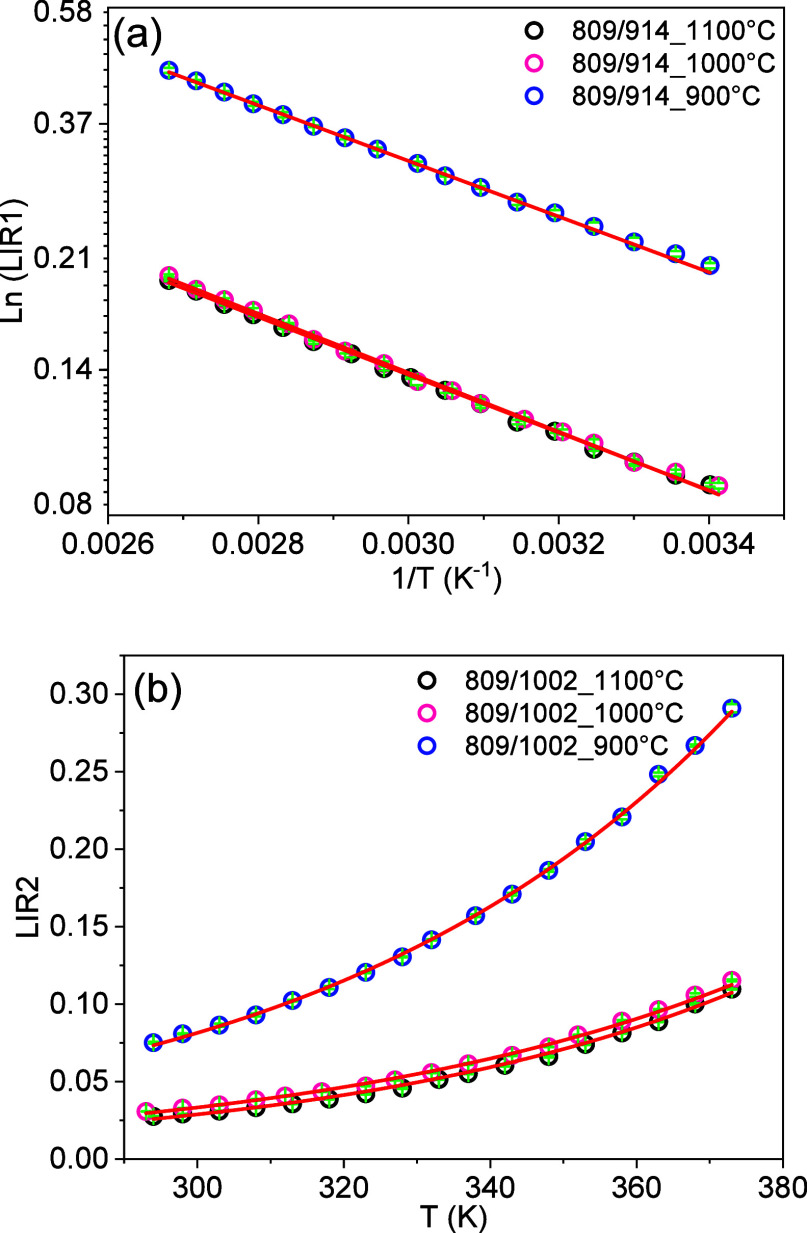
Temperature evolution of the luminescence intensity ratio (a) LIR1 in the monologarithmic scale and (b) LIR2 in the linear scale of the YVO_4_:1%Er,1%Nd sample annealed at different temperatures between 293 and 373 K. The open circles correspond to the data, and the red curves are the fitting using eqs 3 and 4 for LIR1 and LIR2, respectively.

To compare the thermal efficiency of LIR1 and LIR2, the relative sensitivities Sr1 and Sr2 were calculated according to the following^31^ formula:

$$\mathrm{Sr}1=\frac{1}{\mathrm{LIR}1}\frac{d\left(\mathrm{LIR}1\right)}{dT}=\frac{\alpha }{T^{2}}$$ 4

and

$$\mathrm{Sr}2=\frac{1}{\mathrm{LIR}2}\frac{d\left(\mathrm{LIR}2\right)}{dT}=R_{0}$$ 5

The variation of the Sr1 and Sr2 values from 293 to 373 K for the YVO_4_:(1%)­Er,(1%)Nd sample annealed at different temperatures (900, 1000, and 1100 °C) is presented in Figure 9. As can be seen, Sr1 decreases with the temperature, while Sr2 is constant. Indeed, constant sensitivity is an interesting feature for a sensor since that implies a uniform performance of the sensor operating at different temperatures. As can be seen in Figure 9a, the samples calcined at 1100 and 1000 °C (larger sizes) presented the higher values of Sr1. For Sr2, the sample with the largest average size (calcined at 1100 °C) showed the best sensitivity performance.

**Figure 9:**
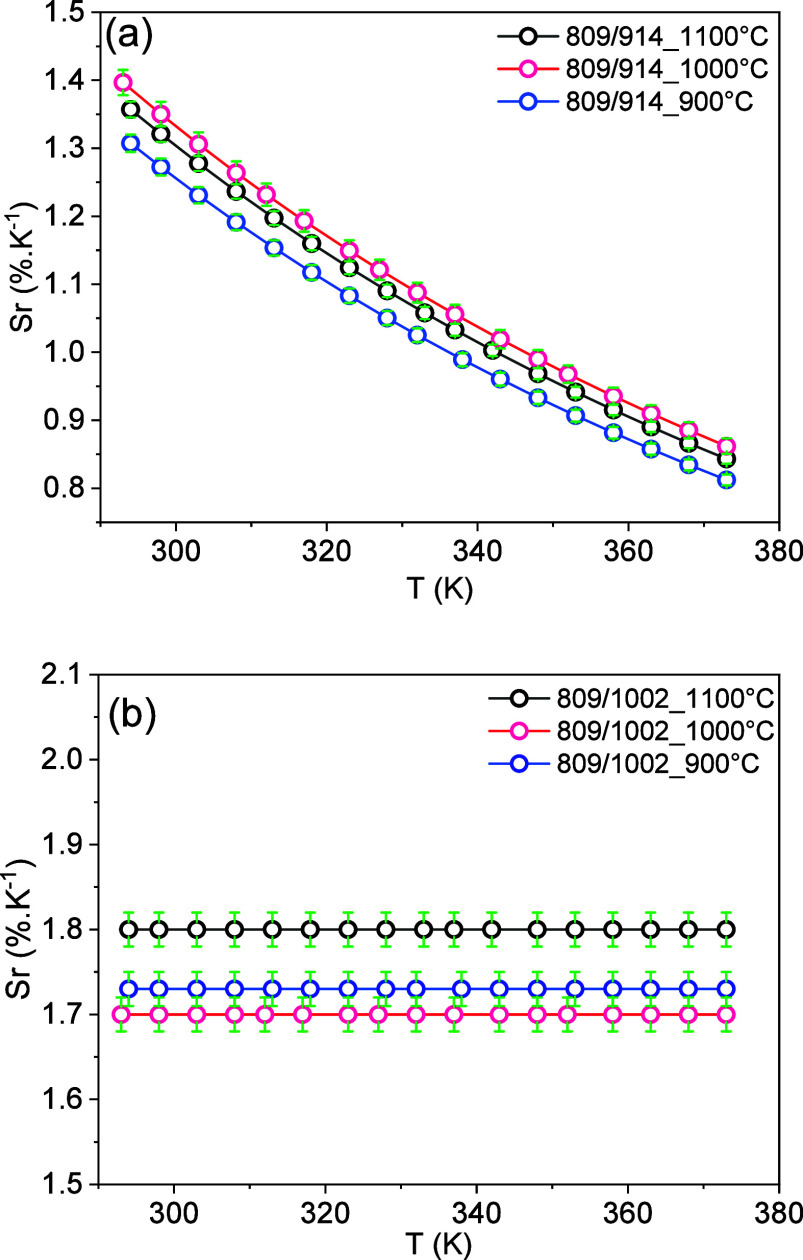
Relative sensitivity Sr of the luminescence intensity ratio (a) LIR1 and (b) LIR2 of YVO_4_:1%Er,1%Nd sample annealed at different temperatures between 293 and 373 K.

It should be noticed that within the experimental error, it is not possible to differentiate the relative sensitivity values between the samples calcined at 1000 and 1100 °C in Sr1. Nevertheless, a clear variation of Sr1 and of Sr2 is observed with increasing particle size, which indicates that for both exploited LIRs, the relative sensitivity is dependent on the particle size. For instance, the highest Sr1 relative sensitivity for the YVO_4_:(1%)­Er^3+^,(1%)­Nd^3+^ calcined at 900, 1000, and 1100 °C temperatures are 1.31 ± 0.01, 1.40 ± 0.02, and 1.36 ± 0.02% K^–1^ measured at 293 K, respectively. On the other hand, the higher values of Sr2 for the YVO_4_:(1%)­Er^3+^,(1%)­Nd^3+^ annealed at 900, 1000, and 1100 °C were 1.73 ± 0.02, 1.70 ± 0.02, and 1.80 ± 0.02% K^–1^ measured at 293 K, respectively. That is, for Sr1, the optimal calcination temperature is 1000 °C and for Sr2, the optimal calcination temperature is 1100 °C. These results reveal how the calcination temperature and, consequently, the particle size could affect the relative sensitivity.

Aiming for practical purposes, any methodology of temperature sensing must exhibit good repeatability and reproducibility. The former is a figure of merit that evaluates the capability of the sensor to provide the same results for repeated measurements under the same conditions. The latter is related to the changes of the same measurement carried out under different conditions.^13,14,33^ The reproducibility analysis for both LIRs were performed, by measuring the LIRs in different days and illuminating distinct portions of the produced powders. The results obtained are summarized in Figure 10. As can be observed, for all samples, different measurements provided essentially the same values of each LIR within the experimental error, which indicates that both LIRs presented a very good reproducibility.

**Figure 10:**
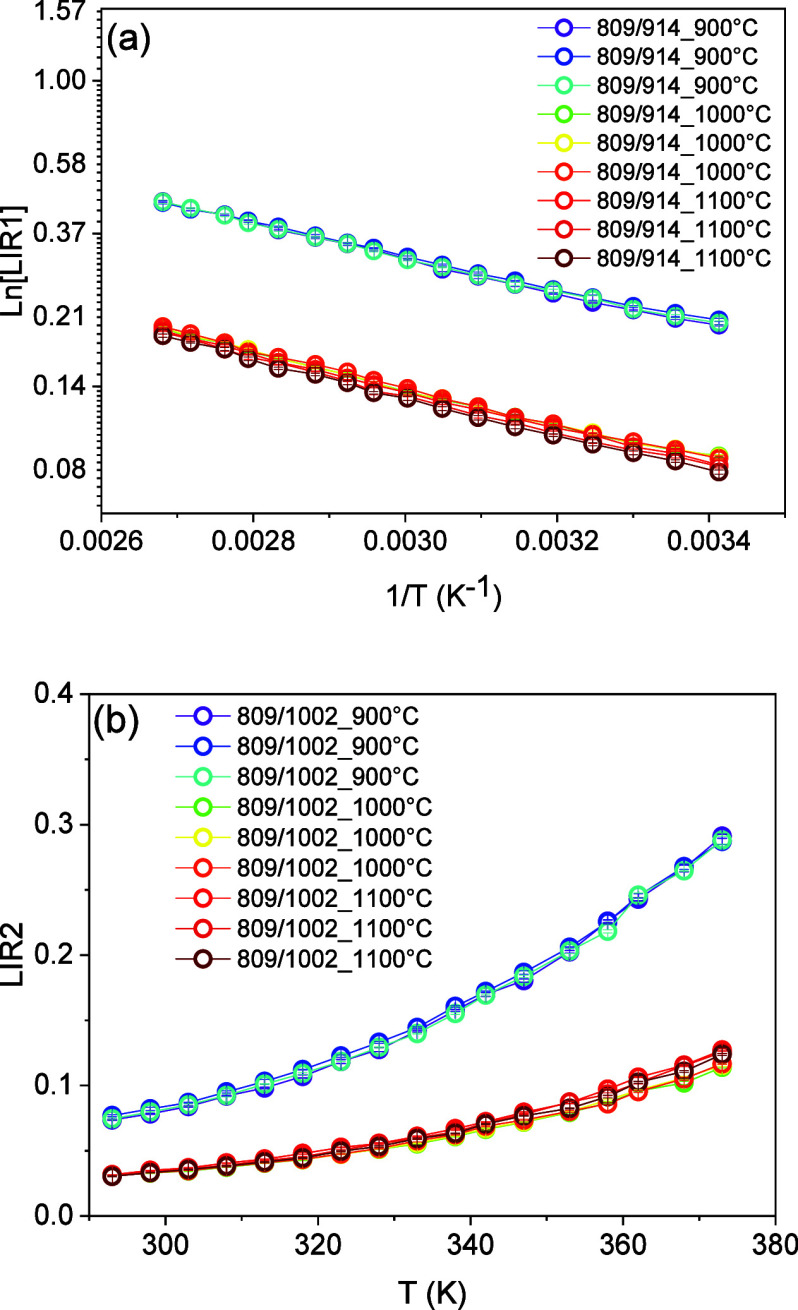
Reproducibility evaluation of (a) LIR1 and (b) LIR2 based on YVO_4_:1%Er,1%Nd.

Another interesting characteristic of a temperature sensor is the temperature resolution or the temperature uncertainty (δ*T*) of the sensor within the investigated temperature range. The temperature uncertainty expresses the smallest temperature variation that can be detected in the experimental procedure.^34^ Considering LIR = *I*_2_/*I*_1_, δ*T* depends on the relative uncertainty of δ­[LIR]/LIR (which is related to the signal-to-noise ratio of the experimental system) and the relative sensitivity.^35,36^

$$\delta T=\frac{1}{\mathrm{Sr}}\frac{\delta \left[\mathrm{LIR}\right]}{\mathrm{LIR}}=\frac{1}{\mathrm{Sr}}\times \left(\sqrt{{\left(\frac{\delta \left[I_{1}\right]}{I_{1}}\right)}^{2}+{\left(\frac{\delta \left[I_{2}\right]}{I_{2}}\right)}^{2}}®\right)$$ 6

Figure 11a and Figure 11b show the behavior of δ*T* as a function of ambient temperature for LIR1 and LIR2 calcined at 900, 1000, and 1100 °C, respectively. As can be seen, the temperature uncertainties associated with LIR1 are around 0.6 K at room temperature and increase little with increasing temperature, remaining below 1 K even at 373 K. The temperature uncertainty associated with LIR2 is below 0.4 K throughout the analyzed temperature range.

**Figure 11:**
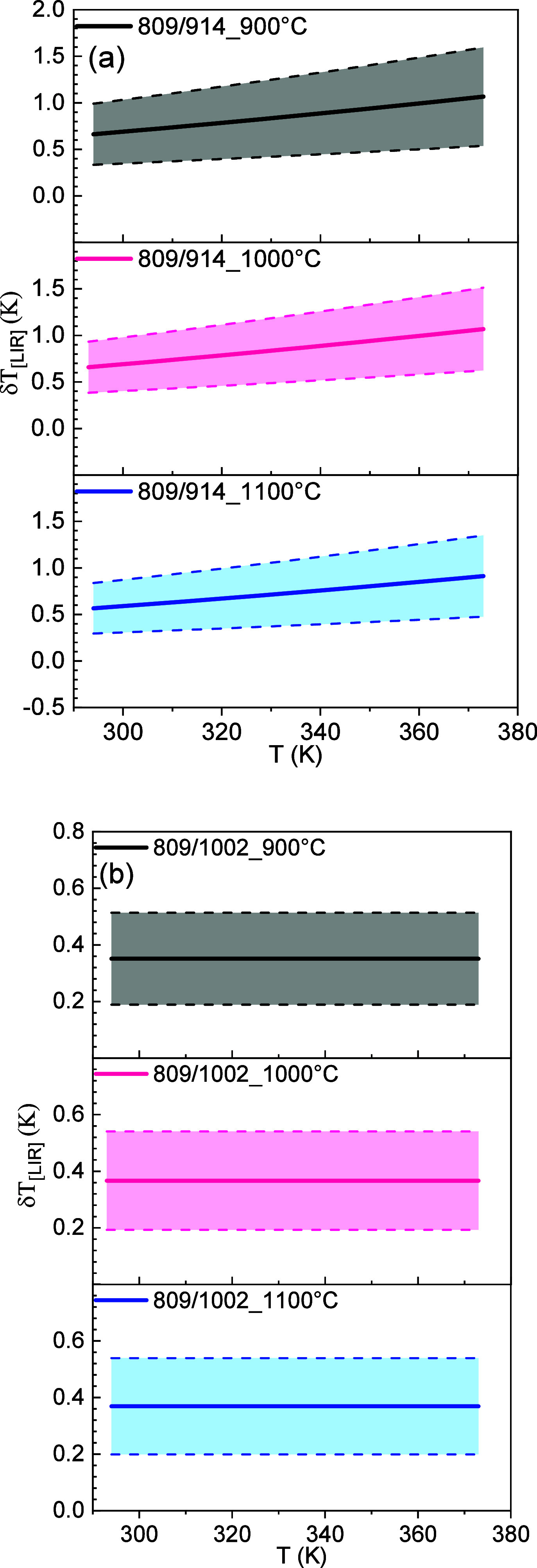
Temperature uncertainty (δ*T*) of the YVO_4_:1%Er,1%Nd samples calcined at 900, 1000, and 1100 °C for (a) LIR1 and (b) LIR2.

At last, it is worth mentioning that the performance of the sensors characterized in the present work are comparable, in terms of sensitivities, with the other sensing schemes based on RE^3+^-doped YVO_4_ and other nanocrystalline hosts already reported in the literature (see Table 2). Nevertheless, in the present study, the excitation and emission wavelengths exploited for thermometry are all within BW I and BW II, which can be advantageous for deep-tissue biological applications.

**Table 2:** Relative Sensitivity (Sr) of Different Ratiometric Luminescence Inorganic Nano- and Submicron Thermometers

material	average size (nm)	ex. (nm)	em. (nm)	**Sr**(%·K^–1^)	ref.
YVO_4_:(1%)Er/(1%)Nd	**262 ± 11**	**660**	**809/914**	**1.40 ± 0.019 @293 K**	**this Work**
YVO_4_:(1%)Er/(1%)Nd	**309 ± 14**	**660**	**809/1002**	1.8 ± 0.02 @293 K	**this Work**
YVO_4_:Nd/Eu	80–90	590	812/700	1.4@299 K	^20^
NaYF_4_:Er/Yb	315 ± 25	975	1010/810	1.05@300 K	^37^
Y_2_O_3_:Er	21	800	522/654	0.87@293 K	^12^
YVO_4_:Nd	N.I.^a^	532	808/880	1.5@300 K	^30^
La_2_O_3_:Yb/Er/Nd	1000–5000	980	825/660	1.7@300 K	^38^
NaYF_4_:Er/Nd/Yb	75	980	742 + 803 + 862/653	1.38@300 K	^39^
YVO_4_:Er	N.I.^a^	280	525/553	1.2@300 K	^3^
Y_2_O_3_:Er/Yb	50	978	539/419	1.57@225 K	^40^
LiBaPO_4_:Nd	30000	800	953/873	1.14@293 K	^14^
YF_3_:Er/Yb	20–100	980	793/840	0.98@293 K	^41^
YF_3_:Yb/Tm	58 ± 10	975	940/650	1.00@305 K	^31^
NaErF_4_	11.6	1530	806/654	0.59@300	^21^
CaO-Y_2_O_3_:Er/Yb	N.I.^a^	980	652/661	0.65@298 K	^42^
YVO_4_:Nd	40–50	808	880/890	0.19@298 K	^43^

a N.I.—not informed.

## 4 Conclusions

In this work, YVO_4_ codoped with 1% Er^3+^ and 1% of Nd^3+^ powdered samples were produced by a modified sol–gel route and submitted to three different calcination temperatures (900, 1000, and 1100 °C). XRD results presented that the obtained codoped YVO_4_ matches with the standard card of tetragonal YVO_4_ (*I*4*1*/*amd*), without the presence of spurious phases. In luminescent thermometry, the emission bands were observed and associated with the transitions from ^4^F_5/2_ and ^4^F_3/2_ to ^4^I_9/2_ and ^4^I_11/2_ of the neodymium ions and from ^4^I_9/2_ and ^4^I_11/2_ to ^4^I_15/2_ of Er^3+^. The performance of this material as a luminescent thermometer was evaluated, and the dependence of its intensities on the temperature variation between 293 and 373 K was investigated. Under visible excitation (660 nm excitation laser), two different LIR methodologies were analyzed. In both cases, light emitted from both dopant species contributed to the overall intensities employed for the LIR. Nevertheless, the first LIR (LIR1) could be empirically modeled by a Boltzmann-like equation, while the second (LIR2) exhibits a single exponential growth behavior. Relative sensitivities up to 1.4 and 1.8% and temperature uncertainties below 1 and 0.4K were achieved employing LIR1 and LIR2 methodologies, respectively. It was also observed that the sensing performance is strongly related to the particle size and LIR scheme. For LIR1, the particles calcined at 1000 °C (∼262 nm of average diameter) exhibit the best sensitivity and uncertainty. On the other hand, using LIR2, the largest particles (calcined at 1100 °C, ∼309 nm average diameter) presented the best temperature sensing performance. The obtained results are particularly very promising, aiming for biological applications in deep-tissue noninvasive thermometry, due to the relatively high sensitivities and low temperature uncertainties achieved and the fact that the excitation and emission wavelengths lie within BW I and BW II.
